# Understanding and Responding to Substance Use and Abuse in the Palestinian Refugee Camps in Lebanon Prior to and During COVID-19 Times

**DOI:** 10.1007/s11469-021-00714-9

**Published:** 2021-11-29

**Authors:** Elie Aaraj, Patricia Haddad, Sara Khalife, Mirna Fawaz, Marie Claire Van Hout

**Affiliations:** 1 Middle East and North African Harm Reduction Association (MENAHRA), Beirut, Lebanon; 2 Faculty of Health Sciences, Beirut Arab University, Beirut, Lebanon; 3 Public Health Institute, Liverpool John Moore’s University, Liverpool, L32ET UK

**Keywords:** Lebanon, Palestinian refugee, Drugs, Displacement, Humanitarian setting, COVID-19

## Abstract

Due to its geographical proximity to the Syrian conflict and the occupied territories, Lebanon has experienced an influx of refugees in recent times. Palestinian refugees are an identified key vulnerable population, with displaced communities increasingly experiencing camp insecurity, vulnerability to drug use and related health harms. A qualitative study consisting of in-depth interviews and focus group discussions (FGDs) was undertaken as part of a regional exercise investigating Palestinian community experiences of substance and drug use in refugee camps. Thematic analysis triangulated the perspectives of 11 professional stakeholders representing United Nations, human rights and non-governmental organizations (NGOs), and eight Palestinian community members. Emerging themes centered on the interplay between socio-economic instability, lack of law enforcement and camp governance contributing to concerning levels of familial, drug and camp violence, trafficking and availability of drugs. Transactional sex and the exploitation of women and children in drug dealing, diversification toward drug manufacture and dealing of drugs with the outside community were described. There is a lack of harm reduction and rehabilitation supports for those in need. This study highlights the complexities in tackling drug dealing and related criminal activity within refugee camps and humanitarian settings, and the vulnerabilities of those living within to harmful drug use.

## Background

The Middle East and North Africa (MENA) region and its ongoing instability and humanitarian crisis in a number of countries, along with the migration and displacement of populations pose multiple challenges for health systems, especially in issues related to HIV and drug use response programs. Repressive and punitive approaches to drugs, criminalization, coercive treatment by the courts and aggressive policing continue to discourage health care seeking by People Who Use Drugs (PWUD), reinforcing marginalization and stigmatization of PWUD and People Who Inject Drugs (PWID) and perpetuating unsafe/high risk use of drugs in the region (Al-Shazly & Tinasti, 2016; MENAHRA, 2021; Sander et al., 2019). In 2019, more than one-third of HIV infections in the MENA region were reported among PWID (UNAIDS, 2020). Refugees and displaced populations face chronic and inter-generational health disparities (Habib et al., 2012; Habib et al., 2014; Habib et al., 2019; Kitamura et al., 2018; MENAHRA, 2021; Van Hout et al., 2020), and are an identified key population in the current Middle East and North African Harm Reduction Association (MENAHRA) situation assessment on drugs and harm reduction in the MENA region (MENAHRA, 2021).

Lebanon due to its geographical proximity to the Syrian conflict and the occupied territories has experienced a substantial influx of refugees in recent times (Thorleifsson, 2016). Refugees make up 30% of Lebanon’s population, the highest concentration per capita of refugees in the world (EU, 2020). Lebanon has been affected by financial crises and countrywide anti-establishment protests, with refugees placing further severe pressure on its economy, infrastructure and health and education services (Shibli, 2014). According to the World Health Organization Regional Office for the East Mediterranean (WHO-EMRO, 2016), refugee numbers have stretched the country’s capacity to respond to mental health, substance/drug use and related infectious diseases. Drugs produced and available in Lebanon include cannabis, heroin, cocaine, amphetamine type stimulants (ATS), synthetic drugs such as Captagon and more recently Salvia (Afsahi and Darwich, 2016; Kerbage & Haddad, 2014; Lebanon Ministry of Public Health, 2017; United States Department of State, Bureau for International Narcotics and Law Enforcement Affairs, 2014; UNODC World Drug Report, 2020). While Lebanon has been classified as a drug transit country, routine surveillance of epidemiological variables such as the rates of substance use disorder, drug-related blood borne viral (BBV) transmission (particularly HIV and hepatitis), and treatment uptake is lacking (Global Initiative against Transnational Organized Crime, 2017; MENAHRA, 2021; Van Hout & Wells, 2016). Available data suggests that general population rates of HIV, hepatitis B and C prevalence in Lebanon are low, but are reported to be rising in refugee populations, and are concentrated among those engaged in drug and sexual risk behaviors (MENAHRA, 2021; WHO-EMRO, 2016).

Of concern is that Palestinian refugees in particular are reported to be ‘*left behind*’ in the official Lebanese policies and strategies and neglected by government public health and drug surveillance (WHO-EMRO, 2016). This is especially the case regarding the 504,000 registered and 75,000 non-registered Palestinians living in twelve refugee camps in Lebanon (Home Office, 2018). They are denied basic civil, political, economic and social rights (i.e. employment, property ownership), have restricted access to public education, health care and social services, and experience acute socio-economic deprivation (Kitamura et al., 2018; UNHCR, 2016). The United Nations Relief Words Agency for the Near East (UNRWA) that operates these camps, and other international aid agencies have reduced reconstruction, and health, education and social services in recent years, despite the rise in camp population. This has resulted in an excessive burden of disease due to trauma, displacement, poverty, malnutrition, violence and congested camp conditions (Habib et al., 2012, 2014, 2019; Kitamura et al., 2018). Multi-morbidities include cancers, cardiovascular, respiratory and mental health disorders, substance abuse and HIV, hepatitis B and C (Habib et al., 2012, 2014).

There has been little progress in activating a joint public health and camp security response to tackle substance/drug use in the refugee camps, with tensions between the ‘*outside*’ (Lebanese community) and ‘*inside*’ (refugees) remaining very high (ANERA, 2019). A climate of instability and drug-related criminal activity heightens camp community vulnerability to substance/drug use and disorders, co-morbidities, high-risk behaviors (i.e. drug injection), BBV transmission and overdoses (UNHCR, 2016; Lamb, 2016; Yamout et al., 2012). In addition to concerns about this threat to communal safety, non-governmental organizations (NGOs) and camp communities fear harmful behavior toward children and stigmatization of PWUD as criminals (Lamb, 2016; UNRWA/UNICEF, 2018).

The 2016–2021 Inter-ministerial Substance Use Response Strategy for Lebanon and the United Nations Office on Drugs and Crime (UNODC) Programme on Drug Control, Crime Prevention and Criminal Justice Reform in the Arab States, categorize Palestinian refugees and displaced persons as a most at risk population (MARP; *Group 2*) due to their marginalization, conflict and displacement-related traumas, and adverse socio-economic situation in the refugee camps (Ministry of Public Health, Ministry of Education and Higher Education, Ministry of Interior and Municipalities, Ministry of Justice, and Ministry of Social Affairs, 2016). Substance/drug use and related harms are context specific and require careful assessment to inform a targeted and community-led response, particularly in humanitarian settings (Ezard et al., 2011; Greene et al., 2019; Green et al., 2018; Kane & Greene, 2018). Enhanced understanding on how drug use is affecting Palestinian communities, especially in light of the additional burdens and lack of services that they are facing is greatly needed (Van Hout et al., 2020). UNRWA, WHO-EMRO, UNODCMENAHRA, the Lebanese Ministry of Public Health, and the Palestinian National Committee on Drugs and Narcotics all recognize this gap and have highlighted the need for reliable strategic information and the development of effective community-led evidence-based initiatives (preventative and support) in the camps.

Our study responds to this need by yielding insights from the community itself about recommendations on how to practically and effectively address these issues from within. It was undertaken in 2021 during the COVID-19 health crisis; which adds an additional layer of understanding with regard to the complexities to addressing the issue of substance use and abuse within the camps during emergency restrictions and contagion.

## Methods

We adopted a critical realist approach (Bhaskar, 1975) which focused on understanding and not merely describing social reality of substance and drug-related activity in the camps, in order to yield insights and to support the generation of community-led responses addressing the issues affecting the camp communities. This qualitative study consisting of in-depth interviews and focus group discussions (FGDs) was undertaken in Lebanon as part of a regional exercise investigating Palestinian community experiences of substance and drug use in refugee camps (Al-Afifi et al., 2019; Van Hout et al., 2019; Wazaify et al., 2020). The design of the data collection instruments was based on extant harm reduction and research expertise of the MENAHRA team in the field, on a systematic review of literature (Van Hout et al., 2019), and on extant published studies using the same methodology and with Palestinian refugees undertaken by the last author in the West Bank and Gaza, and Jordan (Al-Afifi et al., 2019; Wazaify et al., 2020). Interview and FGD guides consisted of questions around perceptions of the changing and current situation regarding substance use, abuse and dependence in the camps; common substances used and the factors contributing to substance use and abuse; impact on families living in the camps; thoughts and opinions regarding potential interventions to address youth and children’s vulnerability to substance use; identified harm reduction, community or psycho-social supports for communities and families affected by the issue; and the impact of COVID-19 on the situation overall. Specific questions included;

**Table:** 

Can you please tell me a little about how long you have lived/worked in the camp? Can you describe the current situation with regard to substance use and abuse in the UNRWA camps in Lebanon? What factors contribute to substance use and abuse in the camp? How does substance use and abuse currently affect Palestinian communities in the camps? What are the needs (related to substance use prevention, social follow-up, and treatment) of Palestinian PWUD and their families/communities in the camps? Are there any services related to drug use that drug users are able to access inside the camps? Outside of the camps? How does the COVID-19 pandemic impact on refugees who use drugs and live in the camp? How can harm reduction NGO and other organizations better support refugees who use drugs and also those in treatment in general, and in particular now given the COVID-19 crisis?	

Ethical approval for the study was granted by Liverpool John Moores University (LJMU), UK, and the Beirut Arab University (BAU) and the Ministry of Public Health in Lebanon. Prior to commencing with the study and subsequent data collection, the MENAHRA team lead (author 1) visited the *Insan* center in *Burj el Barajneh* camp to meet with key informants and gatekeepers. This visit provided insight of the drug use situation in the camps and introduced the MENAHRA team and the research objectives to members of the community. Gatekeepers were advised around the purpose of the study, and where community members volunteered to partake, provided their contact details to the research team.

Data collection was conducted by the MENAHRA team (authors 1 to 3) with a purposive sample consisting of community members living in or close to the Palestinian refugee camps (including former drug users), and key informant stakeholders representing government officials, UN and NGO with professional in-depth knowledge regarding the health situation and drug market dynamics in the refugee camps and from the surrounds. Recruitment of community members was supported by referral of trusted personnel which, in our case, were gatekeepers of the community. In terms of key informant stakeholders, MENAHRA invited government officials, UN agencies and NGO that are known to work within Palestinian camps to participate in the study.

Due to camp and government restrictions during COVID-19, interviews and FGDs were conducted concurrently, via telephone and online using Zoom. In-depth interviews (n = 8) were conducted with male community members; five were former drug users in rehabilitation at the *Insan* Rehabilitation Center at the *Burj Al Barajneh* camp; and three were living within or in close proximity to three camps in the Beirut area; *Burj el Barajneh*, *Shatila* and *Sabra* camps. It was not possible to recruit female community members due to the sensitive nature of the study. Eleven key informant stakeholders were identified as having key informant expertise on the situation in camps close to Beirut and the situation in general for Palestinian refugees in Lebanon (five males, six females). They were consulted using one FGD (n = 9) and two in-depth interviews. The FGD was conducted with representatives from UN agencies and key NGO working to support the camps (ANERA, UNICEF, Tajamoh NGO, Jana NGO, Nabaa NGO, Al Najda NGO, and Witness Association for Human Rights) and who operate programmes in *Ain El Helwe*, *Shatila* and *Burj Al Barajneh* camps. Two in-depth interviews were conducted with the Directors of the National Mental Health Program at the Lebanese Ministry of Health and the *Insan* drug rehabilitation center at the *Burj Al Barajneh* camp.

All participants were informed about the study aims and objectives prior to data collection and were given the opportunity to ask questions regarding participation prior to agreeing to partake. Informed consent was provided prior to voluntary participation. Participants were requested not to mention any names or personal information including their own. Participants did not receive any incentive or compensation for participation. Interviews and FGDs were facilitated by authors 1, 2 and 3. No identifying information such as signatures or personal information was collected in order to protect participant confidentiality and privacy. All participants were allocated a code prior to the interview/FGD. Identifiers that inadvertently appeared in audio recordings were removed within 24 h of the interview/focus group. All interviews and FGDs were audio recorded, with detailed note taking, transcribed and translated into English using a back translation method.

The data was analyzed using thematic analysis (Braun & Clarke, 2006; Braun et al., 2019) which was deemed appropriate to garnering an in-depth understanding of the complexities and dynamics around substance and drug use in the camps. This approach consists of several key steps to ensure scientific rigor by the team (authors 2–4): reading and re-reading the transcription, individually and in pairs to share and identify early ideas; the development of coding schemes and the systematic coding of data by two members of the team; an iterative process to organize codes into groups in developing themes and subthemes; team refinement and review of generated themes as a collective and with examination of coherence of patterns across themes; and finalization of themes and the naming of themes. As patterns and outliers emerged, periodic briefing sessions were held between all authors (1–4), and all members of the team assisted in interpretation of the narratives, reaching consensus around identified themes, and clarifying ambiguities in the data. We added a further layer of triangulation of sources in terms of perspectives across community member and key informant professional stakeholder narratives when raising the abstraction level.

## Results

Seven themes emerged from the data analysis: *Poverty and unemployment*; *Hopelessness and drug related coping*; *Inner and outer camp aspects of lawlessness*; *Drug availability, user and dealer networks*; *Impacts of COVID-19 on drugs and drug use*; *Drug related violence and exploitation of the vulnerable*; and *Imperatives for community support.* The results centered on the socio-economic contextual aspects underpinning vulnerability and drug-related activity based on the interplay between poverty, isolation, and lack of law enforcement and camp governance. This contributed to troubling levels of familial, drug and camp violence; production, trafficking and availability of drugs; transactional sex and the exploitation of women and children in drug dealing; and diversification toward drug manufacture and dealing drugs with the outside community. A lack of harm reduction and rehabilitation supports for those in need was mentioned. We use illustrative quotes to represent and articulate the essence of each theme, where appropriate.

Across the three Palestinian refugee camps that were visited by researchers, both community members and key informant stakeholders underscored how the current socio-economic instability, along with poverty and lack of camp governance and law enforcement both within and outside the refugee camps acted as drivers of drug-related activity (drug use, dealing, drug-related violence, criminal activity and exploitation of the vulnerable). A key informant stakeholder summed this up by stating; ***“… drugs have affected the reality of the Palestinian communities in the camps, which also in itself increased drug use.” (Stakeholder #7, male).***

### Poverty and Unemployment

Involvement in drug-related activity in the camps appears to be underpinned by the lack of rights to employment (and property ownership) in the country, consequent extreme poverty and lack of opportunity to support families in their basic needs. In particular, participants mentioned how unemployment in Palestinian youth contributed to a lack of empowerment and subsequent engagement in both drug-related criminal activity and harmful drug use. A community member observed; ***“Those who use drugs are not in the right state of mind…the bigger portion of drug users in the camps lack jobs, freedom, dreams and aspiration. If someone studied and graduated, where would they work? Sometimes you might see someone using drugs in the camp and you would pity them… some people say it is a pity that they earned degrees and are still using drugs…why? Because they can’t find a job… they are hopeless.” (Community Participant #6).***

A key informant stakeholder reported how for many (PWUD and community members) engagement in the trafficking and sale of drugs became normalized and legitimized over time as source of income to support families; ***“Housewives are considering it a source of income and one woman was saying she works with her son in law because they need to make a living… and now because they are considering it a source of income, they have found a justification for it.” (Stakeholder #S8, female).***

### Hopelessness and Drug-Related Coping

All community members remarked that the economic crisis affecting Lebanon, the denial of their civic rights to gain employment by the Lebanese government, and their refugee status inhibited their integration into Lebanese society. This lack of civic engagement and feeling of detachment from the host society was described by all as contributing to the precipitation of hopelessness, depression, boredom, and the justification of drug use and participation in the sale of drugs to others. A community participant mentioned; ***“Unemployment, sitting at home all day, boredom, and nothing to do… and family problems.” (Community Participant #5).*** They described how drugs are used as coping mechanism and as ways/strategies to escape from the daily realities of life in the camps; ***“Every person does this to run away from the problems in their life…We cannot blame them, every person needs something to deal with reality” (Community Participant #5).***

This common observation regarding the changing dynamics of drug use and entrenched drug-related activity was supported by a key informant stakeholder who said; ***“…Even more so with drugs, other than the methods of use that have changed, the justifications have changed; camps closed, deteriorating economic situation… all the misery that is gathered within the camps and the drugs become a way for running away and forgetting issues…” (Stakeholder #S1, male).***

### Inner and Outer Camp Aspects of Lawlessness

Participants illustrated lawlessness within the camps by mentioning the lack of action and by the absence of inner camp governance, where all participants (community members and key informant stakeholders) indicated how drug dealing was conducted in plain sight of the authorities. This was observed to fuel normalization of drug dealing and the accessibility and availability of drugs in the camps. Two community participants illustrated this; ***“Selling drugs is public and ordinary, like shops that sell vegetables so it makes it very available. Those who have the intention to use will find it no matter if it was inside or outside the camp… they will find it when they look for it…plus in the camps there isn’t a presence of Lebanese law enforcement, so it’s very available inside the camps…” (Community Participant #7)*** and ***“…. There is no one to regulate or enforce the law and there is no presence of Lebanese law enforcement. When something is a taboo and prohibited by the government and inside the camps it available, no one will say no…” (Community Participant #3).***

Some professional stakeholders described the overt presence of clandestine drug manufacture operating within the camps; and serving the local Lebanese communities living on the fringes. A female key informant stakeholder commented; ***“… we hear of factories… everything is exposed and you don’t need an investigation, just talk to anyone at the camp… there are differences between camps but the common thing is that the issue is so public that even children are aware of it… drug dealers and promoters are not hidden… they don’t care, they just do it… They are also suppliers for communities outside of the camp and not just the camp.” (Stakeholder #9, female).***

### Drug Availability, User and Dealer Networks

Participants observed how the aforementioned socio-environmental aspects of camp life underpinned by community desensitization toward drug dealing has contributed to widespread availability of inexpensive drugs including pharmaceuticals such as Tramadol and cough syrups, solvents, illicit street drugs (heroin, hashish) and salvia, with the use and abuse among a host of diverse demographic groups, including youth, women, young girls and children. Several community member and key informant stakeholders illustrated this; ***“…drugs are widespread and available in cheap prices… the danger lies in the fact that drugs are available in very cheap prices and so are accessible to a large group of youth… they are using cough syrups, thinner through sniffing… chemicals that are very dangerous to health.” (Stakeholder #1, male)*** and ***“… all available, all the products come in the knowledge of the government and no one asks questions…” (Community Participant #4).***

All key informant stakeholders indicated how there are known places for obtaining drugs, and that people in the camp community are protecting their locations leading to the spread of awareness and use of drugs. Many voiced their concern around widespread marketing and promotion of drugs in different settings, including at school gates. The exploitation of children as drug runners was described as an increasing concern. Several key informant stakeholders described this and said; ***“The danger is that there are networks that are using young children for drug promotion. I know about a case of a 12-year-old child who was being used as a distributer within the camp due to the financial issues and this is the worst form of child labour…” (Professional Stakeholder #1, male)*** and ***“…We have youth promoting drugs at school gates… we have teenage girls… 14–15 years of age that say they took these pills by mistake through their friends.” (Stakeholder #7, male).***

Key informant stakeholders stated that NGOs involved in community and school–based drug prevention in the refugee camps had become a threat to drug traffickers and to their source of income; ***“There are big entities that are promoting drugs through children. There were women threatening us to not talk about drug prevention within the camps. The situation is now worse due to the security situation.” (Stakeholder #7, male).*** Participants noted that a variety of inexpensive drugs were available in the camps, and were particularly accessible to young people and children. A stakeholder observed that; ***“… One gram of heroin for 20,000 Lebanese pounds [approx. 13 US dollars], so the situation is very bad. There are many dangers for teenagers and especially for schoolchildren who can get an envelope for 1500 Lebanese pounds [approx. 1 US dollar]… Farawla [Tramadol] is very trendy among them… and salvia [hallucinogenic plant]…” (Stakeholder #7, male).***

### Impacts of COVID-19 on Drugs and Drug Use

With regard to the impact of the COVID-19 health emergency, both community members and stakeholders observed that threat of contagion in the camps did little to stem drug-related activity, and where in some instances drug dealing became more public in the camps, and harmful drug use was perceived to have increased. A key informant stakeholder said; ***“…it’s on the rise… it’s getting worse by the day. What everyone is saying is true. Last year and the year before the situation was not as bad. It’s becoming worse. It wasn’t this normalized. There used to be drugs but there was taboo surrounding this issue. The spread this year is unprecedented…” (Stakeholder #4, male).***

Awareness around the risks of community transmission of COVID-19 was low, and according to one community participant did little to impact on drug availability and overt drug dealing (often through windows) in the camps; ***“… none of the drug users are aware of Corona, they are not aware of anything of their surroundings… nothing has changed… all business as usual, all is available… they dispense it through a window… it is everywhere you go… those who use drugs know where to find it…” (Community Participant #1).*** Other community members observed illicit drug initiation by individuals in the refugee camps during the pandemic; ***“…some people who never had drugs have started using hashish…” (Community Participant #3).***

### Drug-Related Violence and Exploitation of the Vulnerable

Many participants observed a steady rise in drug related crime (theft, muggings), leading to a volatile unstable and unsafe environment for those living in the camps. These crimes were described as underpinned by both the necessity to generate income by selling stolen goods; and by efforts to stave drug-related withdrawals. This was illustrated by a community participant who said; ***“…there were muggings, there was an incident where a woman was buying a pack of cigarettes and she got mugged… drug usage and dealing has caused a lot of mugging and other kinds of crime… they use the stolen goods to sell and buy drugs later with the money… like stealing a motorcycle to sell and buy the needed dose of drugs…” (Community Participant #2).***

In addition, the drug abuse situation was described by many key informant stakeholders as giving rise to intense family conflicts and varied forms of inter-familial violence (gender based, physical and sexual violence), sexual and child exploitation of family members, transactional sex and aggression all arising from the need to afford the drugs. Several professional observations were made; ***“… break up of families, familial violence, gender violence related to women and girls, child labour, sex work… I was surprised when I was hearing these stories, but it was all shown through numbers… Violence in all its forms… when they were asked why they are using kids, they said that these are not kids they’re 12 so they are old enough…” (Stakeholder #2, female)*** and* ‘I hear more about networks for women for sex work, but I also hear about men engaging in sex to be able to afford drugs. We encountered a case where a young adult; 21 years of age who stabbed his brother because he wanted drugs” (Stakeholder #6, female).*

In addition to small violent incidents caused by drug users themselves, there were reports of gang or faction activity where drug dealers and drug dealing networks fought in public, using guns and opening fire in public. All community member participants described how they and their families feared for their lives; ***“ … there are a lot of issues that rise from such things… in one of the streets twenty people died due to altercation including a very recent incident that a woman died with a child in her hand… there was a violent conflict related to drug deals, and no one could do anything about it” (Community Participant #5).*** The realities of threatening behavior, protection racketeering and the undercurrent of tension in community life caused by drugs and drug-related networks are palpable in the camps. Observations were made from community and key informant stakeholders working in the camps; ***“…People cannot speak about the issue or their life would be threatened… so drug dealers do what they like down here… you cannot do anything about it, we just feel sad for our friends that have fallen for this lifestyle… some dealer groups also make regular people from the community pay money for protection, otherwise they would attack people’s shops and threaten their livelihoods… they also have become a source of power and authority, for instance if I need something from you I can use them as leverage to get what I want…” (Community Participant #8)*** and ***“…creation of armed groups outside of the law… they are protecting themselves and harming others. These armed groups are harming the camp community.” (Stakeholder #3, female).***

Armed groups were observed by many participants (both community and key informant stakeholders) to protect and encourage drugs and weapons trafficking (including by children) in the camps, and transactional sex of both genders; ***“Use of children in armed conflicts was also seen a lot. There is a connection when drug dealing is the second biggest business after arms dealing…” (Stakeholder #7, male)*** and ***“Regarding sex work, in one of the camps there are two known places, and some say that they are protected by armed groups… For men there are no specific places where it is conducted like for women. The men just talk and make arrangements between each other…” (Stakeholder #3, female).***

### Imperatives for Community Support

Community members emphasized their need for social support, particularly in the form of funding for community-strengthening strategies aimed at restricting drug use and trafficking, generating employment, building community engagement, supporting families and youth, and increasing awareness of drug harms. Community members made several recommendations; ***“…social and psychological support is the base, providing jobs and alternatives to be productive in the society…families need awareness on how to deal when there is a drug user in their household… the media never show how to know that your son is using drugs, they only shed a light that there is a problem but never address effective information that people need to know to break the stigma…” (Community Participant #1)*** and ***“…We need awareness campaigns, on the dangers of drugs, financial support, clothes, food… since poverty is what makes people use drugs… offering jobs, since it’s very hard… those who don’t have degrees cannot find jobs at all and those who have degrees rarely find jobs, in areas very far away” (Community Participant #5).***

The need for free and accessible drug treatment and rehabilitation services was acknowledged by some community members, who stressed the lack of community and individual awareness about pathways toward rehabilitation. This was supported by key informant stakeholder perspectives which underlined the deficit of drug prevention, treatment and rehabilitation services and supports for those affected, both users and their loved ones inside the camp confines. Those seeking help are faced by financial difficulties in covering costs of treatment and rehabilitation provided by Lebanese NGOs outside of the camps. Aftercare inclusive of family interventions was also observed to be lacking for those on treatment completion. The revolving door of relapse was described by many on return to life in the camps. A range of illustrations regarding the lack of drug rehabilitation and targeted family supports were made; ***“…the camp could benefit from rehab centers that don’t charge fees to rehabilitate… after someone is rehabilitate and goes back hope, families should learn how to deal with their son and not make him feel abandoned or cast out…” (Community Participant #2)*** and ***“…some are helping with referrals, but when they go to external centres and then return to their environment and friends that have facilitated drug use… it’s like we didn’t do anything. Some people that have been in rehab relapsed because they came back to the environment that they were in. There needs to be cost free centres so that people can afford to go…” (Stakeholder #5, female).***

A key informant stakeholder emphasized the need for a comprehensive multi-stakeholder plan involving all camp stakeholders underpinned by initiatives tackling drug demand (camp governance hand in hand with Lebanese authorities) and with buy-in from the camp communities themselves in order to support change, along with the need for drop-in centers, and those providing aftercare services to support rehabilitation. He illustrated his point as follows; ***“…You cannot put a plan in place without people from the community such as stakeholders, different NGOs, partners, political parties… there should also be a link with the law with the Lebanese government. If the law is not by your side, it will not be effective. Prevention, awareness, and other interventions are important but you need the law to be by your side. All the stakeholders within the camps should be a part of a comprehensive plan starting with a study like this, so that everyone assumes their responsibilities, and then the activities and interventions would be handled by NGOs – it’s the easiest thing to put activities, but you cannot put aside the economic and security situation.” (Professional Stakeholder #1, male).***

## Discussion

This is the first qualitative study examining the perspectives and experiences related to substance use of the Palestinian population living in refugee camps in Lebanon. Findings echo that of similar studies conducted in the Palestinian refugee communities in the West Bank, Gaza and Jordan (Al-Afifi et al., 2015, 2019; Damiri, 2019; Damiri et al., 2018; Glick et al., 2018; Massad et al., 2016; Van Hout et al., 2019; Wazaify et al., 2020).

Our study highlights the substantial impact of drug activity and drug use on Palestinian refugees, their families and communities, notwithstanding the existing health, social, political and economic disadvantage (trauma, lack of property and employment rights, illnesses and psychological distress) they encounter and navigate when displaced into overcrowded and sub-standard housing in the Lebanese camps. The Palestinian community living in the camps has been, and is still burdened by many challenging situations including poverty, unemployment, lack of services, the spread of radical religious groups, violence and drug dealing both inside and outside the camps, among many others. Palestinians in Lebanon are not permitted to be employed in a number of sectors, which creates economic instability and a lack of civil commitment. Emerging themes centered on the interplay between poverty, socio-economic instability and the lack of law enforcement and camp governance. This contributes to concerning levels of familial, drug and camp violence, trafficking and availability of drugs, transactional sex and the exploitation of women and children in drug dealing and diversification toward dealing drugs with the outside Lebanese community. There is a lack of harm reduction and rehabilitation supports for those in need.

Similar to extant literature from other countries hosting Palestinian refugees, our study illustrates the tensions within the camps and between camp refugees and the outside community, along with factors related to drug use such as stress, trauma and refugee camp insecurity and vulnerability to drug use and related health harms (Afifi et al., 2019; ANERA, 2019; Damiri, 2019; Massad et al., 2016; Syam et al., 2019; Thabet & Dajani, 2012; UNRWA/UNICEF, 2018). In 1969, after what was called *“The Black September”*^1^ in Jordan, the Palestinian Liberation Organization (PLO) and other armed groups took over the security responsibility in the camps, inhibiting the access of Lebanese law enforcement to camps. Neither Lebanese officials nor Palestinian camp authorities can ignore issues related to security such as lawlessness, camp governance, and the criminal networks involved in drugs, human and weapons trafficking. As mentioned in our study, addressing rates of drug use and achieving demand reduction warrants close cooperation between the Lebanese law enforcement and those in charge of camp governance. Tackling the camp-community, and indeed cross border criminal networks, engaged in drug production, transit and dealing poses urgent security challenges for the Lebanese authorities, exacerbated by political and economic instability (Global Initiative against Transnational Organized Crime, 2017).

Our study illustrates how the closed nature of the refugee camps fuels violence along with drug and criminal activity within, and supports diversification of trade between the populations inside and outside the camps. Normalization of drug activity appears to have developed over time, with drug dealing conducted in plain sight of camp authorities. Given the absence of Lebanese law enforcement, community members whose safety is at risk are left with little recourse to address drug activity in the camps. Of concern is the exploitation of vulnerable individuals, particularly the dealing and use of drugs by women and children in the camps. The exploitation of children at school gates and the presence of a women’s network promoting drug use and stifling attempts to raise awareness around the harms of drug use is deeply concerning.

Furthermore, there is an urgent need to respond to the substantial vulnerabilities of those living with harmful drug use, and of the Palestinian individuals, families and communities affected by the harms related to drug use and dependence. The situation of Palestinian refugees in the Lebanese camps is compounded by lack of access to prevention, harm reduction and drug treatment and rehabilitation services for at risk and affected individuals and their families. Although there are numerous NGOs working with the Palestinian community, both inside and outside the camps, there is no specific program on drug awareness or prevention, and there are no free services provided. Moreover, in some places, NGOs are not allowed to focus on this topic due to security reasons. When funds are available UNRWA provides financial support for those seeking detoxification. With the exception of one small center (*Insan*) in *Burj el Barajneh* camp (see UNRWA, 2017), there is a complete absence of accessible drug information, education, prevention, harm reduction, family support and treatment initiatives within or near the twelve camps (Ministry of Public Health, 2019). The *Insan* center is in need of technical assistance and professional capacity building for team members in order to comply with the accreditation system of the Lebanese Ministry of Public Health (MoPH). Further, while they are recognized by government as a vulnerable key population in the 2016–2021 Inter-ministerial Substance Use Response Strategy for Lebanon, Palestinian refugees lacking financial ability, and having their civil rights restricted cannot access existing government drug disorder treatment structures.

The connection between the camps and local Lebanese communities cannot be underestimated. Substance use and abuse is a rising public health concern outside the camps, among the general Lebanese population, due to increasing prevalence, earlier ages of initiation, high rates of drug injection, the presence of co-morbidities, and the increased threat of several diseases related to high-risk sexual activity and unsafe injecting among drug users (Karam et al., 2019; Lebanon MoPH, 2017; MENAHRA, 2021). Women with substance use disorders and minors are identified by the Lebanese government as key populations requiring additional supports due to the significant increase in the number of females receiving drug disorder treatment (Lebanon MOPH et al., 2016; 2017; 2019). Restrictions to camp access further obstruct Lebanese government attempts to conduct routine health surveillance particularly that of BBV transmitted through risky sexual activity and injecting drug use. Such impediments also create challenges for researchers, NGOs and other interested parties.

The bridge of transmission of disease connecting the camps, their fringes and the Lebanese population cannot be underestimated. While little is known with regard to the prevalence of HIV and other viral diseases inside camps, the convergence between drug use and high risk sexual activity among drug users or those exploited by family members for drug money in the camps is concerning. Our study reveals high-risk sexual activity within transactional sex involving men and women in the camps. On the outside, there is an observed convergence of drug and sex–related risk behaviors that increase the risk of HIV among men who have sex with men (MSM) and refugee populations in Lebanon (Heimer et al., 2017). Also, while less than 3% of the HIV epidemic in Lebanon is attributed to unsafe injecting of drugs, accurate data is not available regarding levels of injecting, numbers of people who inject drugs (PWID) and their HIV/Hepatitis status in the general community (Larney et al., 2020; Ministry of Public Health, 2016). While daily injecting rates are estimated to be relatively high in Lebanon (Colledge et al., 2020), with PWID having an average age of 29.5 years (Hines et al., 2020), closed small networks among Lebanese PWID appear to reduce transmission risks (Mahmud et al., 2020). Given the close nature of the refugee camps, this may very well be the case for Palestinian PWID. It is therefore encouraging that with regard to HIV treatment, the Lebanese Ministry of Public Health is providing high quality, 100% free of charge treatment to all the Lebanese PLHIV including Syrian, Palestinian or other settled refugees.

Stigma and the complexities around gaining access to camps hamper our understanding of high and hidden risk behaviors in the camps, and the ability for NGOs to provide harm reduction and other supports for those affected and their families. There is a remarkable lack of knowledge on drug use and harmful or dependent use among the families in the camps. In line with the UNODC and WHO International Standards for Treatment of Drug Use Disorders (UNODC/ WHO, 2020) and the International Standards on Drug Use Prevention (UNODC, 2018), a comprehensive health, education, social and legal action plan should be developed and implemented. This plan should cover law enforcement, prevention and awareness (including parental and family skills, youth resilience and youth protection from exploitation), harm reduction strategies and screening for BBV/sexually transmitted infections. It should also support the detoxification and rehabilitation of PWUD. In order to leverage a community led success, this comprehensive plan should be generated from within, and involve all stakeholders in the camps, including the camp governance in order to leverage a community led, culturally and contextually relevant response, and work closely with Lebanese authorities in the surrounds (Van Hout et al., 2020). With the support of UNRWA and the MoPH, the care providers in the camps need to develop a referral system and to build a network with all concerned centers and NGOs, inside and outside the camps, to offer the community of PWUD and those in need of support a wide wrap-around range of services related to drug use, spanning prevention, harm reduction, treatment, rehabilitation and aftercare. This effort must be inclusive of the media, which can support in spreading awareness and targeted messages. Such targeted and community-led responses are recommended in refugee and humanitarian settings (Ezard et al., 2011; Greene et al., 2019; Green et al., 2018; Kane & Greene, 2018).

Lastly, the impact of the COVID-19 pandemic and the resulting restrictions did little to impede the continued operations of drug dealing, and drug use, including new initiatives in the camps. Our study reveals that there is lack of awareness regarding COVID-19 transmission and related disease mitigation among the Palestinian community in the camps in general, and more specifically among PWUD. This is troubling, especially given the growing literature on the unique vulnerabilities of PWUD/PWID during COVID-19 pandemic times; including those living in camps in the MENA region (Van Hout et al., 2021).

### Strengths and Limitations

The contribution of the present article is twofold; it contributes to the small and developing field of research on drug use in Palestinian refugee camps in the Middle East and it presents the views and perspectives of those who work with the community and those who live in the Palestinian refugee camps in Lebanon. The validity of the study itself is supported by using a critical realist approach which focuses on understanding, and not merely describing social reality of drug use in the camps, and by the triangulation across data sources, methods and the extensive literature review. We note the limitations of this small scale yet important study. Narratives may have been affected by the lack of face to face data collection.

## Conclusion

Due to its geographical proximity to the Syrian conflict and the occupied territories, Lebanon has experienced an influx of refugees in recent times. Palestinian refugees are an identified key vulnerable population, with displaced communities increasingly experiencing camp insecurity, vulnerability to drug use and related health harms. Our study highlights the legal and public health complexities involved in addressing drug dealing and related criminal activity within the Palestinian refugee camps and humanitarian settings in general; the way strategies to achieve this goal are hampered by the closed nature of these contexts; and the substantial vulnerabilities of those living within the camps to harmful drug use. We reveal the tensions related to camp security, along with those arising between the populations inside and outside the camps. It underscores the need for high level discussions within UNRWA, the main service provider in the camps to assume the responsibility for their role in bringing together all concerned stakeholders to design and implement a comprehensive response plan for substance use within the camps. Such a plan which would include prevention, rehabilitation, and harm reduction aspects would require legitimacy if proposed through UNRWA. Efforts to create a comprehensive substance use response plan in line with that of the Lebanese National Mental Health Program would help to address the issue and facilitate applying existing policies for services (such as opioid agonist treatment) that do not exist within the camps.

## Footnotes

1 The black September is known as the battle between the Jordanian army and the troups of the Palestine Liberation Organization (PLO) who were trying to gain power and authority in Jordan. This was followed by the Arab leaders summit where a convention named the “Cairo Agreement” was signed allowing the PLO to have local authority at the Palestinian camps in Lebanon, and giving them the right to fight against Israel from within the Lebanese territories (Cairo Agreement, 1969).
